# Crude subcellular fractionation of cultured mammalian cell lines

**DOI:** 10.1186/1756-0500-2-243

**Published:** 2009-12-10

**Authors:** Paul Holden, William A Horton

**Affiliations:** 1 Research Center, Shriners Hospitals for Children, Portland, Oregon 97239, USA; 2 Department of Molecular and Medical Genetics, Oregon Health Sciences University, Portland, Oregon 97239, USA

## Abstract

**Background:**

The expression and study of recombinant proteins in mammalian culture systems can be complicated during the cell lysis procedure by contaminating proteins from cellular compartments distinct from those within which the protein of interest resides and also by solubility issues that may arise from the use of a single lysis buffer. Partial subcellular fractionation using buffers of increasing stringency, rather than whole cell lysis is one way in which to avoid or reduce this contamination and ensure complete recovery of the target protein. Currently published protocols involve time consuming centrifugation steps which may require expensive equipment and commercially available kits can be prohibitively expensive when handling large or multiple samples.

**Findings:**

We have established a protocol to sequentially extract proteins from cultured mammalian cells in fractions enriched for cytosolic, membrane bound organellar, nuclear and insoluble proteins. All of the buffers used can be made inexpensively and easily and the protocol requires no costly equipment. While the method was optimized for a specific cell type, we demonstrate that the protocol can be applied to a variety of commonly used cell lines and anticipate that it can be applied to any cell line via simple optimization of the primary extraction step.

**Conclusion:**

We describe a protocol for the crude subcellular fractionation of cultured mammalian cells that is both straightforward and cost effective and may facilitate the more accurate study of recombinant proteins and the generation of purer preparations of said proteins from cell extracts.

## Findings

### Background

The expression of recombinant proteins in cultured cell lines has become an increasingly valuable tool for the study of protein trafficking and function. In many or most instances it is desirable to purify the recombinant protein to allow accurate study of such functions. While this can be complicated enough for a protein that is secreted into the culture medium of the cell system, it becomes even more daunting when the protein of interest resides within the cell, perhaps in a single specific compartment. In this case it is desirable to be able to isolate the target protein with minimal contamination by other proteins, potentially from compartments that the protein of interest may never encounter. In most standard cell lysis procedures a single lysis buffer, perhaps NP40 or RIPA is used to generate a total cell lysate essentially comprising a soup of proteins and creating abundant opportunity for non specific interactions.

Another consideration that arises when one is studying a mutant protein is the question of whether or not the protein is soluble in the extraction buffer used and thus whether or not all of the protein has been harvested, enabling an accurate assessment of the effect of the mutation on the intracellular trafficking and function of the protein. The use of more than one single lysis buffer can help to address this issue, particularly if the stringency of the additional lysis buffer is higher, thus increasing the chances of solubilizing any misfolded mutant proteins.

Additionally, many proteins do not reside permanently within a given intracellular compartment but rather shuttle between compartments to carry out their functions e.g. various transcription factors that shuttle between the cytosol and the nucleus. A simple subcellular fractionation protocol that allows easy and reproducible generation of fractions representative of specific subcellular organelles would facilitate the study of such dynamic changes in intracellular protein localization.

Here we present a simple protocol for the isolation of crude subcellular compartments from cultured mammalian cells, sequentially generating fractions enriched for cytosolic, membrane bound organellar, nuclear and insoluble proteins respectively.

### Optimization of digitonin concentration for extraction of cytosolic proteins

The primary step of the protocol that we have developed is the permeabilization of the plasma membrane using digitonin to effectively release the contents of the cytosol. Digitonin binds to and forms pores in membranes by complexing with membrane cholesterol and other β-hydroxysterols [1]. The extent of binding and permeabilization thus depends upon the accessibility of the membrane and its sterol composition [2]. At low concentrations of digitonin the cholesterol rich plasma membrane can be effectively solubilized with little to no solubilization or permeabilization of intracellular membranes that are lower in cholesterol such as the endoplasmic reticulum (ER) and mitochondrial membranes. Similar concentration dependent effects on the degree of cell lysis by digitonin have also been shown in studies on parasites [3,4].

**Figure 1:**
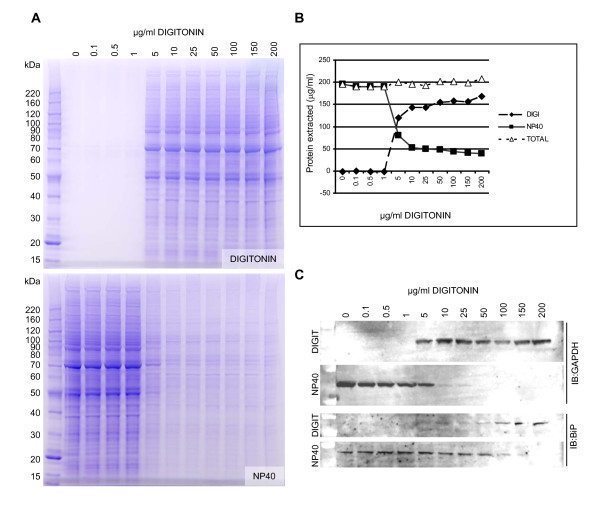
**Optimization of digitonin concentration for the extraction of cytosolic proteins**. (A) HEK293 cells were plated at a density of 4 × 10^5 ^cells per well of a 12 well plate and were harvested 48 hours later. Cells were then lysed in 400 μl of buffer containing digitonin at the concentration indicated. Following centrifugation and collection of the supernatant the remaining cell pellet was further extracted in NP40 lysis buffer. An aliquot of each extract was then analysed by 4-12% SDS PAGE (Invitrogen #NP0322) followed by staining with Coomassie blue. (B) The concentration of protein in each extract was determined by colorimetric protein assay (Pierce #23232) and the results were plotted to demonstrate that total protein extracted was the same regardless of the starting concentration of digitonin. (C) Extracted proteins from each fraction were analyzed by Western blot using an anti GAPDH antibody (NOVUS Biologicals #300-221B) as a marker of the cytosol and an anti BiP antibody (SIGMA # G918) as a marker of the endoplasmic reticulum.

To optimize this primary step we tested a range of concentrations of digitonin from 0 to 200 μg/ml on Human Embryonic Kidney (HEK293) cells, a cell line widely used in the production of recombinant proteins [5]. Briefly, cells were cultured in 12 well plates and were harvested by trypsinisation followed by gentle lysis in buffer containing digitonin at a specified concentration. Following digitonin extraction, the remaining cell pellets were then lysed in NP40 lysis buffer to solubilize intracellular membrane bound organelles such as the endoplasmic reticulum (ER). Samples were then analyzed by 4-12% SDS-PAGE followed by staining with Coomassie blue or by Western blotting using antibodies to the cytosolic protein GAPDH and the ER lumenal chaperone protein BiP/grp78 (Figure 1). Using this approach it was determined that at least 5 μg/ml digitonin was required to release the contents of the cytosol from HEK293 cells, as judged by Coomassie blue staining (Figure 1A) and colorimetric assay of protein concentration in the extracts (Figure 1B). At this concentration a significant amount of GAPDH was further extracted by the NP40 buffer indicating incomplete cytosolic extraction. Conversely at concentrations of 50 μg/ml digitonin or higher some BiP was also extracted indicating partial lysis of the endoplasmic reticulum (Figure 1C). Thus it was decided that a concentration of 25 μg/ml digitonin was optimal, giving near complete extraction of the cytosol as indicated by GAPDH levels, with negligible permeabilization of the ER membrane, as indicated by BiP levels. Following this starting concentration of digitonin, the ER was then fully extractable using NP40 lysis buffer. It is worth noting that regardless of the starting concentration of digitonin in each case, the final total amount of protein extracted remained the same indicating that the initial digitonin extract did not have any refractory effects on the extractability of proteins in the subsequent step (Figure 1B).

### Solubilization and extraction of nuclei and insoluble proteins

**Figure 2:**
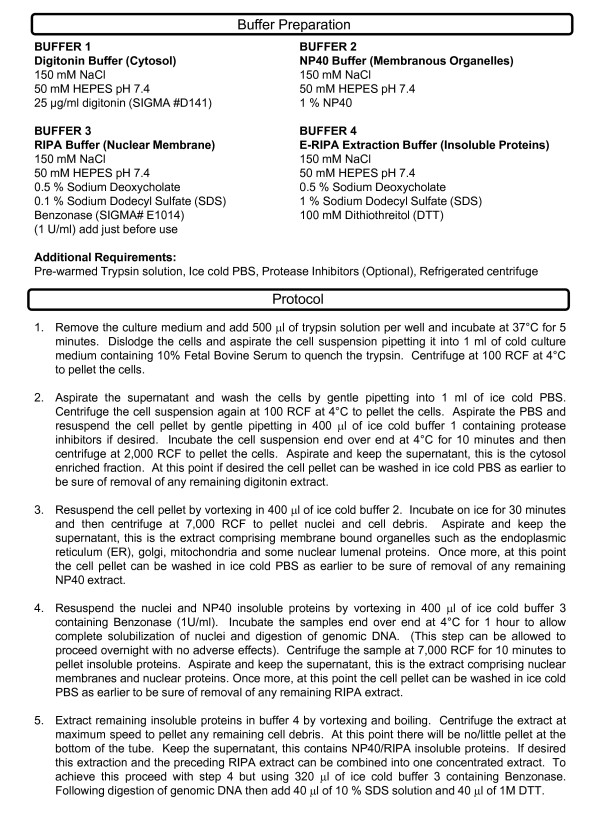
**Crude subcellular fractionation protocol**. Recipes to prepare the required buffers are given at the top of the figure along with additional requirements for the protocol. The protocol is then listed step by step below.

**Figure 3:**
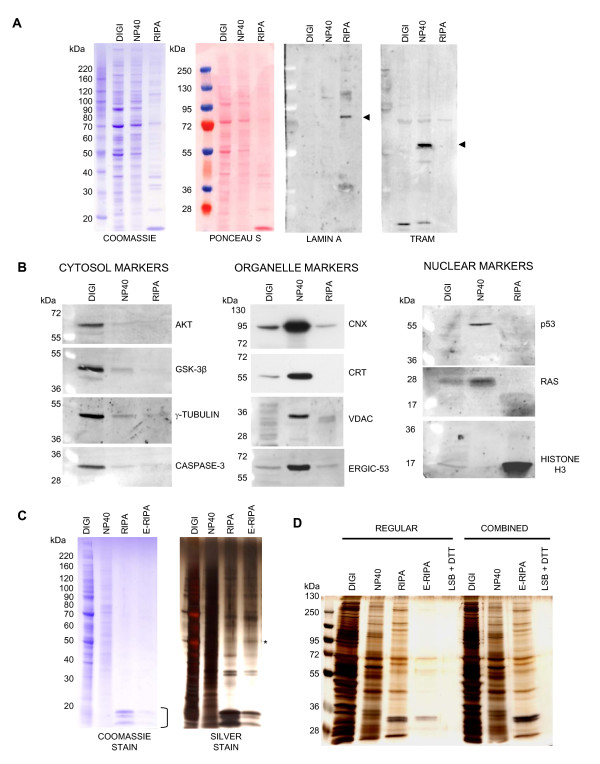
**Solubilization and extraction of nuclei and insoluble proteins**. (A) HEK293 cells were plated at a density of 4 × 10^5 ^cells per well of a 12 well plate and processed according to the protocol in Figure 2. An aliquot of each extract was then analyzed by 4-12% SDS PAGE followed by staining with Coomassie blue or by Western blotting and probing with antibodies to the nuclear membrane protein Lamin A (SIGMA #L1293) or the ER membrane protein TRAM both highlighted by arrowheads. (B) Extracts generated in (A) were analyzed by 8 or 12% SDS PAGE followed by Western blotting and probing with antibodies to various cytosolic; Akt, Cell Signaling Technology (CST #9272), GSK-3β, (CST #9315), Caspase-3 (CST #9662) and γ-Tubulin (Santa Cruz Biotechnology, sc-7396), Organellar; Calnexin (SIGMA #C4731), Calreticulin (Stressgen #SPA-061), VDAC/Porin (SIGMA #V2139) and nuclear; p53 (CST #9282), RAS (Upstate Biotechnology #05-516) and Histone H3 (ABCAM #ab1791) proteins as indicated or with anti V5 antibody (Invitrogen #R96025) in the case of ERGIC-53. (C) Equal volumes of extracts were analyzed by 4-12% SDS-PAGE followed by staining with Coomassie blue and then silver staining. The bracket alongside the Coomassie stained gel shows the position of Histones in the RIPA nuclear extract and the asterisk next to the silver stained gel denotes a protein band unique to the E-RIPA extract. (D) The subcellular fractionation protocol was carried out in full and in duplicate. In one experiment the RIPA and E-RIPA extracts were prepared as a single extract as described in step 5 of the protocol (Combined). In both cases following the E-RIPA extract any remaining pellet was extracted by boiling in LSB + DTT.

The remainder of the subcellular extraction protocol, shown in its entirety in figure 2, was then applied. Briefly, following the NP40 lysis step the nucleus was solubilized and its contents released using RIPA buffer supplemented with Benzonase (SIGMA) to digest DNA and RNA. As shown in figure 3A the RIPA extraction successfully solubilized the nuclear membrane as indicated by the presence of Lamin A, a nuclear membrane protein in the RIPA extract that was not evident in any of the preceding extracts. Also shown in this figure is the presence of the ER membrane protein TRAM entirely in the NP40 extract. As further demonstration of the distinction between fractions we tested a panel of antibodies to various cytosolic, ER, mitochondrial and nuclear proteins. As shown in figure 3B the cytosolic proteins AKT, GSK-3β, γ-Tubulin and Caspase-3 are found almost exclusively in the digitonin extract. Similarly the ER resident chaperones Calnexin and Calreticulin are found predominantly in the NP40 extract, as is the mitochondrial membrane protein VDAC/Porin. We also transfected cells with a construct encoding V5/6×-HIS epitope tagged ERGIC-53, a membrane component of the ER-golgi intermediate compartment and this localized almost exclusively to the NP40 fraction. Finding nuclear proteins that localized predominantly to the RIPA fraction was more challenging. The transcription factors p53 and RAS were found in the NP40 and digitonin/NP40 extracts respectively. It is to be expected that subcellular distribution of transcription factors would change depending upon the cell cycle phase since all are initially synthesized in the cytosol and traffic to the nucleus to function when required. The fact that no p53 or RAS were evident in the RIPA extract may be indicative of their absence for functional reason or of permeabilization of the nuclear membrane by NP40. Histone H3 however was found exclusively in the RIPA extract. Taking these facts together it is perhaps more appropriate to state that extraction with NP40 solubilizes membrane organelles such as the ER, Golgi and mitochondria and may also permeabilize the nuclear membrane. Complete solubilization of the nuclear membrane and some nuclear resident proteins however requires RIPA buffer as evidenced by the presence of Lamin A and Histone H3 in this extract.

Following extraction in RIPA buffer the final extraction in enhanced RIPA buffer (E-RIPA) solubilized any remaining proteins as shown in figure 3C by Coomassie stain and subsequent silver stain. At least one distinct protein band is present in the E-RIPA extract relative to the preceding RIPA extract demonstrating extraction of previously insoluble protein. The Coomassie stain also shows the presence of Histones in the nuclear extract, as later confirmed by Western blotting. While no pellet was visible following the E-RIPA extraction, addition of 1× Laemmli sample buffer to the tube followed by boiling and analysis by SDS-PAGE and silver staining showed that no proteins remained after the E-RIPA extraction (Figure 3D). Also shown in this figure is the combinatorial approach from step 5 in the protocol where the RIPA and E-RIPA extracts are generated as one single concentrated extract, again showing that no proteins remain after these steps.

### Demonstration of the advantages of crude fractionation over whole cell lysis and scale up of the approach

**Figure 4:**
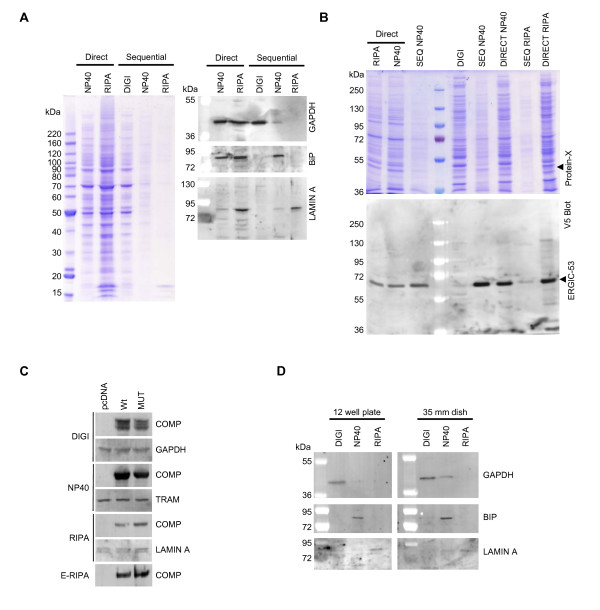
**Demonstration of the advantages of crude fractionation over whole cell lysis and scale up of the approach**. (A) HEK293 cells were plated at a density of 4 × 10^5 ^cells per well of a 12 well plate and subsequently lysed directly in NP40 or RIPA lysis buffer or processed according to the protocol in Figure 2 (Sequential). An aliquot of each extract was then analyzed by 4-12% SDS PAGE followed by staining with Coomassie blue or by Western blotting and probing with antibodies to various marker proteins of specific intracellular organelles as previously. (B) HEK293 cells were plated at a density of 4 × 10^5 ^cells per well of a 12 well dish and then transfected the next day in triplicate with 400 ng of a construct encoding V5/6×-HIS tagged ERGIC-53. Cells were extracted 48 hours later either directly in RIPA buffer, directly in NP40 buffer or according to our extraction protocol. Proteins were then partially purified from each extract using Nickel resin (SIGMA #P6611) and eluted proteins were analyzed by 10% SDS PAGE followed by staining with Coomassie blue or by Western blotting and probing with the anti V5 antibody. (C) HEK293 cells were plated at a density of 4 × 10^5 ^cells per well of a 12 well dish and then transfected the next day in with 1600 ng of pcDNA6/V5-His (Invitrogen) or constructs encoding WT or MUT COMP. Cells were harvested 48 hours later and extracted according to our protocol. (D) HEK293 cells were plated at a density of 4 × 10^5 ^cells per well of a 12 well dish or 8.4 × 10^5 ^cells per well of a 35 mm dish and processed according to the protocol in Figure 2. An aliquot of each extract was then analyzed by 4-12% SDS PAGE followed by Western blotting and probing with antibodies to GAPDH, BiP and Lamin A.

To demonstrate the advantage of stepwise extraction of proteins from cultured cells over whole cell lysis, we took HEK293 cells in culture in a 12 well dish and lysed one well directly in 400 μl of a typical NP40 lysis buffer, one in 400 μl of a standard RIPA lysis buffer and another according to our stepwise procedure. As shown in figure 4A by staining with Coomassie blue, direct lysis in either NP40 or RIPA buffer generates a more complex sample creating opportunities for potentially non specific protein interactions, whereas a stepwise lysis approach generates less heterogeneous samples enriched for particular organelles. Western blot analysis shows that the recovery of representative markers of each compartment appears to be approximately equal indicating no loss of protein or reduction in yield due to the stepwise approach. Interestingly, NP40 does not appear to solubilize the nuclear membrane of HEK293 cells since no Histones are evident in the Coomassie stained gel and minimal Lamin A staining is observed by Western blot.

As proof of concept of the application of this method in the generation of purer preparations of recombinantly expressed proteins, we transiently transfected HEK293 cells with a construct encoding V5/6×-HIS tagged ERGIC-53. Cells were then either lysed directly in NP40 or RIPA buffer or were extracted sequentially according to our protocol. Subsequent analysis of extracts by SDS-PAGE followed by staining with Coomassie blue or by Western blotting and probing with an anti-V5 antibody demonstrated the advantages of crude subcellular fractionation over direct lysis. As can be seen in figure 4 purification of ERGIC-53 from the NP40 extract obtained in the stepwise procedure using Nickel Resin resulted in a purer preparation and higher yield of protein than from direct NP40 or RIPA lysis due to less interference by proteins from the cytosol becoming non-specifically bound to the resin/ERGIC-53.

To illustrate our concept that not all proteins are recovered in a single lysis step, particularly when overexpressed in a recombinant system, we transfected cells with constructs encoding WT and mutant (MUT) cartilage oligomeric matrix protein (COMP) using lipofectamine 2000 (Invitrogen) as per manufacturer's instructions [6]. COMP is a secreted glycoprotein of the extracellular matrices of various skeletal tissues [7]. As shown in figure 4C the majority of the protein resides in the NP40 extracted ER fraction undergoing synthesis and trafficking. However a significant amount of protein, particularly in the case of MUT COMP, was recovered in the E-RIPA extract indicative that it was misfolded and aggregated (Holden et al, manuscript in preparation). Thus our approach allows for the complete extraction of recombinantly expressed proteins which may be critical in determining functional consequences of mutations in any given protein.

We also considered that since some experimental approaches require significant amounts of material, it would be desirable to scale up this approach accordingly. As such we calculated buffer volumes required based on surface area of the culture vessel used as shown in Table 1, assuming a proportional increase in cell number. Testing of this scale up to the level of a 35 mm dish showed that the fractionation efficiency was maintained (Figure 4D).

**Table 1:** Volumes of buffer required for protocol scale up according to culture vessel surface area.

Culture Vessel	Surface Area (cm^2^)	Buffer Volume (ml)
100 mm dish	55	5.8

60 mm dish	21	2.2

35 mm dish	8	0.84

6 well plate	9	0.95

12 well plate	3.8	0.4

24 well plate	2.0	0.2

### Application of the approach to other cell lines

**Figure 5:**
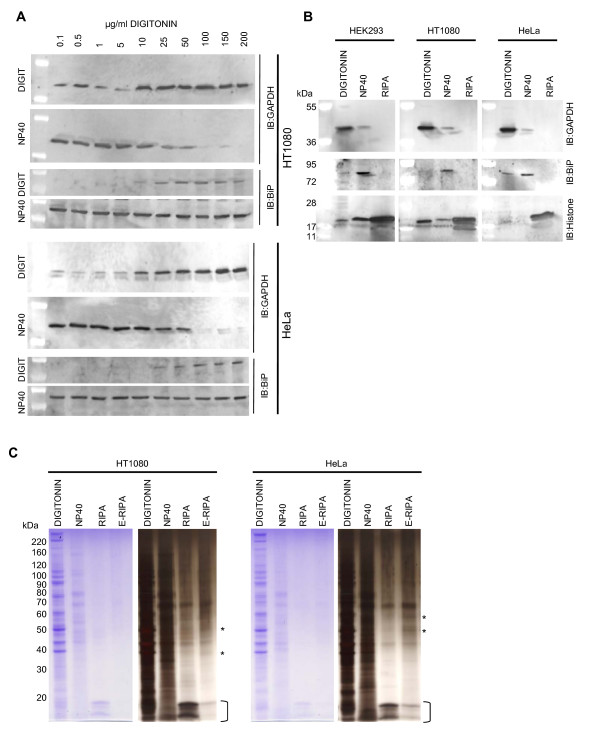
**Application of the approach to other cell lines**. (A) HT1080 cells (2 × 10^5^) and HeLa cells (1 × 10^5^) were plated in the wells of a 12 well plate and the digitonin concentration required for optimal extraction of the cytosol was determined as in Figure1. (B) The remainder of the protocol from Figure 2 was then applied using 100 μg/ml digitonin as the optimal cytosolic extract in each case. An aliquot of each extract was then analyzed by 4-12% SDS PAGE followed by Western blotting and probing with antibodies to GAPDH, BiP and Histone H3 (Used as an alternative nuclear marker due to weakness of the Lamin A signal in HeLa cells). (C) Samples were analyzed by 4-12% SDS PAGE followed by staining with Coomassie blue and then silver staining showing the difference in protein composition of each sample. The bracket alongside the gels marks the position of Histones in the RIPA nuclear extract and the asterisk next to the silver stained gels denotes protein bands unique to the E-RIPA extracts.

To ensure that this approach was not only applicable to HEK293 cells we repeated the procedure for two other commonly used human cell lines, HT1080 [8] and HeLa [9] cells beginning with the digitonin optimization step for the extraction of cytosolic proteins. As shown in figure 5A the concentration of digitonin required for successful extraction of cytosolic proteins with minimal perturbance of the integrity of the ER was between 25 and 100 μg/ml for each cell line. It is also worth noting that both HT1080 and HeLa cells displayed a greater sensitivity to lysis by digitonin, some GAPDH being extractable even at 0.1 μg/ml digitonin whereas HEK293 cells required at least 5 μg/ml. This is not surprising given the mechanism of action of digitonin on cell membranes and the natural variance in membrane sterol composition of between 10 and 20% that occurs between different cell types. In the application of this technique to a given cell line, the decision regarding the optimal concentration of digitonin to use for the initial step will ultimately depend upon the desired outcome i.e. whether one desires total extraction of cytosolic proteins and can tolerate some contamination from partial lysis of other compartments, or whether one requires a partial cytosolic extract essentially free from contamination. Additionally, the concentrations of digitonin can be optimized within a narrower range following an initial experiment such as this in order to maximize distinction between fractions.

Continuation of the protocol using 100 μg/ml digitonin for the cytosolic extraction in each case demonstrated that, as was the case for HEK293 cells, distinct fractions comprising membrane bound organellar proteins, nuclear proteins and insoluble proteins could also be generated for HT1080 and HeLa cells (Figures 5B and 5C). Due to the weakness of the signal from Lamin A we chose to use an alternative marker of nuclear proteins, Histone H3 (ABCAM) which clearly localized predominantly to the nuclear extract. Once more, the silver stained gels in figure 5C reveal the presence of unique proteins in the E-RIPA extracts that are not present in the preceding RIPA extract.

## Discussion

We have developed a simple, inexpensive protocol for the isolation of crude subcellular fractions of cultured cell lines. The protocol uses simple buffers and equipment and requires only approximately 2 hours from start to finish. While here we have presented data from three commonly used human cell lines, HEK293, HeLa and HT1080, we anticipate that the technique can be applied to any cell line in culture with some optimization of the initial digitonin extraction step.

We envisage several useful applications of this technique; the generation of purer preparations of recombinant proteins from cultured cells, more thorough analysis of trafficking and secretion of wild type and mutant proteins and simplification of protein extracts for proteomic analysis.

The generation of purer preparations of recombinant proteins from cultured cells is highly desirable. One such advantage of having a purer preparation would potentially be in the context of the study of protein-protein interactions. Taking our expression and purification of ERGIC-53 as a example, if one was to attempt to study the interactions of this protein with other ER/Golgi proteins, our stepwise fractionation approach of essentially removing the bulk of cytosolic proteins prior to lysis of the ER, would potentially minimize non specific interactions between ERGIC-53 and cytosolic proteins. While we do not demonstrate this directly, figure 4B shows an approximately 50 kDa protein (Protein-X) that appears to co-purify with ERGIC-53 using our sequential lysis approach. While this band is also apparent when direct NP40 and RIPA lysis approaches are used, there are clearly other similar sized proteins also present which may complicate the identification of this band.

In the second case, studies of mutated recombinant proteins via transfection of cultured cell lines can often be misleading. In many cases, protein mutations lead to aberrant folding which can in turn lead to intracellular aggregation and insolubility in typical cell lysis buffers such as NP40. Using the approach described here, essentially all proteins from within a cell are effectively solubilized by use of buffers of increasing stringency, thus helping to ensure that every folded intermediate of a given protein is extracted which may help to give a more accurate picture of the effect of a mutation on the fate of a protein. We have ourselves, as shown briefly, recently used this approach in the study of mutations in cartilage oligomeric matrix protein that cause the dwarfing condition pseudoachondroplasia (Holden et al, manuscript in preparation).

While we are aware that the use of digitonin and detergent fractionation in the preparation of subcellular extracts is not a novel concept, as far as we are aware our protocol as presented here represents the first open access protocol that encompasses simple stepwise subcellular fractionation of cultured mammalian cells in totality, giving a complete extract of all cellular proteins. Given its simplicity, reproducibility and cost effectiveness we feel that this protocol will be extremely useful to scientists across a broad range of disciplines, particularly given its accessibility to all in this format.

## List of abbreviations

NP40: nonyl phenoxylpolyethoxylethanol; RIPA: Radio immunoprecipitation assay; HEK293: Human embryonic kidney 293; ER: Endoplasmic reticulum; DIGI: Digitonin; VDAC: Voltage Dependent Anion Channel; ERGIC: ER to golgi intermediate compartment; COMP: Cartilage oligomeric matrix protein; LSB: Laemmli sample buffer; CNX: Calnexin; CRT: Calreticulin.

## Competing interests

The authors declare that they have no competing interests.

## Authors' contributions

PH was responsible for the design and implementation of the experiments in this study and preparation of the manuscript. WH was responsible for supervision, discussion and manuscript review. Both authors read and approved the final manuscript.
